# Supplementary material for: Design and Analysis of a Single System of Impedimetric Biosensors for the Detection of Mosquito-Borne Viruses
Source: Biosensors (Basel). 2021 Oct 7;11(10):376. doi: 10.3390/bios11100376 (PMC8533959; doi:10.3390/bios11100376)
Supplement: Supplementary file 1 [file biosensors-11-00376-s001.zip › biosensors-1393282-supplementary.pdf]

Supplementary Material

Design and analysis of a single system of impedimetric biosensor for the detection of mosquito-borne viral diseases

Fahmida Nasrin[1], Kenta Tsuruga[2], Doddy Irawan Setyo Utomo[3], Ankan Dutta Chowdhury[1], Enoch Y. Park[1,2,3*]

1 Laboratory of Biotechnology, Research Institute of Green Science and Technology, Shizuoka University, 836 Ohya, Suruga-ku, Shizuoka 422-8529, Japan

2 Laboratory of Biotechnology, Department of Agriculture, Graduate School of Integrated Science and Technology, Shizuoka University, 836 Ohya, Suruga-ku, Shizuoka 422-8529, Japan

3 Laboratory of Biotechnology, Graduate School of Science and Technology, Shizuoka University, 836 Ohya, Suruga-ku, Shizuoka 422-8529, Japan

* Correspondence: park.enoch@shizuoka.ac.jp

Table S1. (a) Circuit diagram for the electrochemical fitting, (b) The electrochemical parameters of the sensor electrode obtained from impedimetric circuit diagram.

(a)

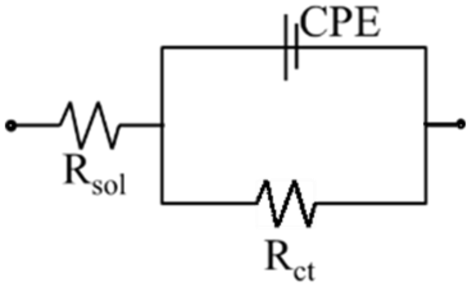

(b)

		R_{sol} (Ω)	CPE (10^{-7} F)	n	R_{ct} (Ω)
**Au-PAni/N,S-GQDs**	0	20.4	5.40	0.345	5837
**CHIKV**	10 fg mL^{-1}	21.2	6.35	0.348	6873
	100 fg mL^{-1}	23.4	6.22	0.342	7925
	1 pg mL^{-1}	27.5	5.12	0.370	9284
	10 pg mL^{-1}	27.7	4.97	0.492	15052
	100 pg mL^{-1}	28.1	6.87	0.542	22944
	1 ng mL^{-1}	31.6	5.68	0.563	35632

The synthesis and TEM images of AuNP-PAni and N,S-GQDs:

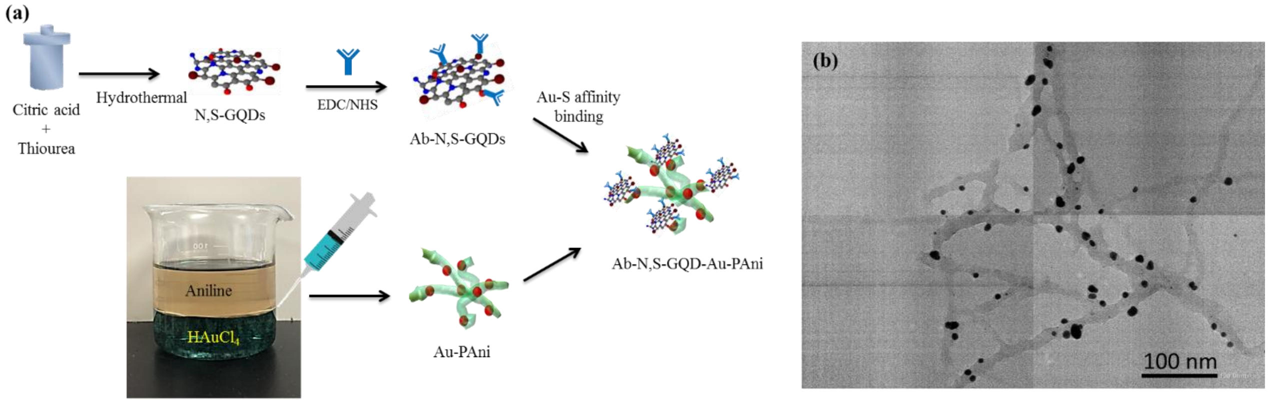

Figure S1. (a) Schematic illustration of Au-PAni-N,S-GQD-Ab sensor electrode, (b) TEM image of Au-PAni nanocomposite.

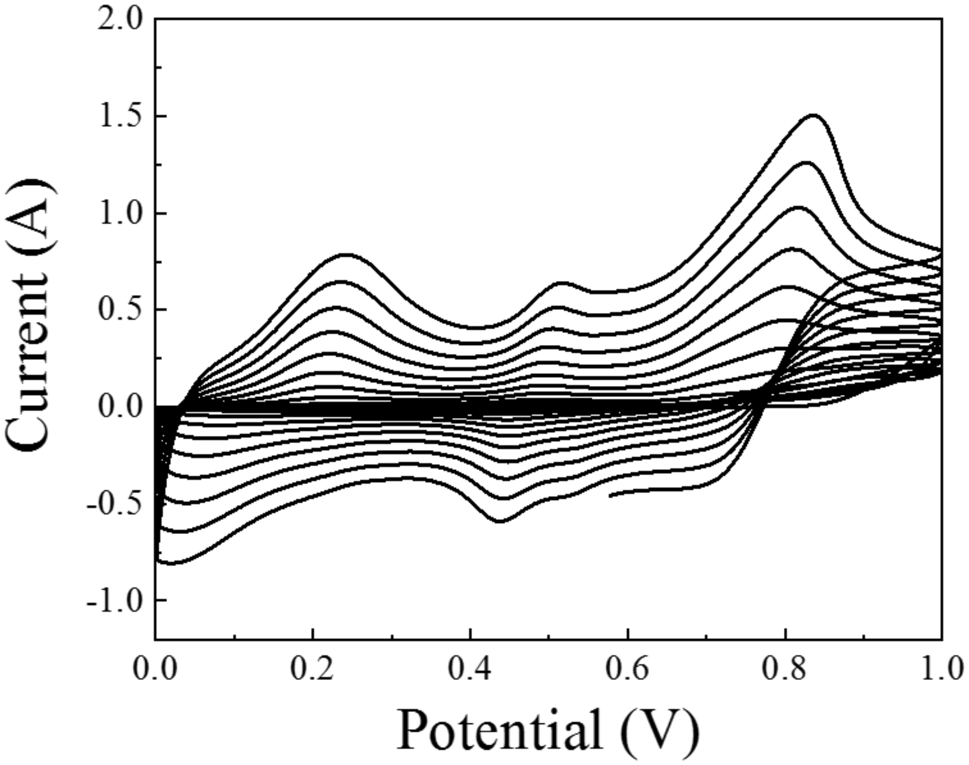

Figure S2. Electropolymerization of polyaniline on the bare Au electrode.

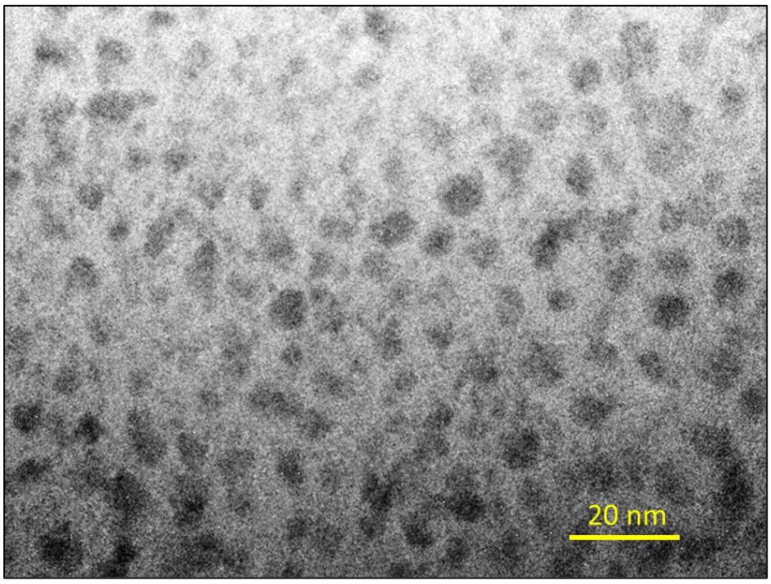

Figure S3. TEM image of N,S-GQDs. The small black particles with a size of 8–12 nm represent the GQD structures.

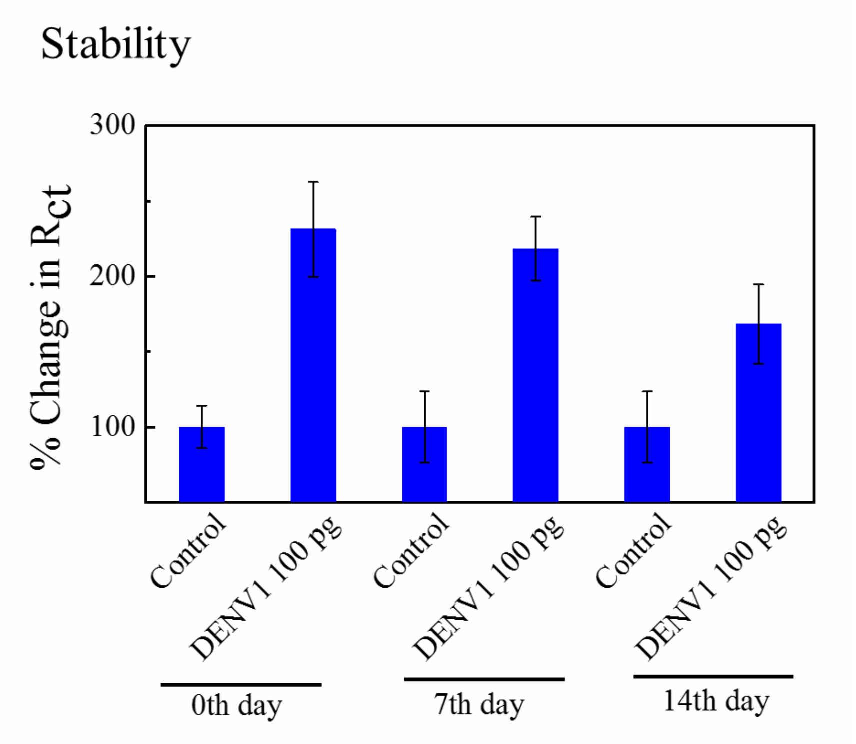

Figure S4. Stability test of the sensor electrodes.
